# Heterogeneity of CEACAM5 in breast cancer

**DOI:** 10.18632/oncotarget.27778

**Published:** 2020-10-27

**Authors:** Marc B. Bechmann, Andreas V. Brydholm, Victoria L. Codony, Jiyoung Kim, René Villadsen

**Affiliations:** ^1^Department of Cellular and Molecular Medicine, Faculty of Health and Medical Sciences, University of Copenhagen, Copenhagen, Denmark; ^2^Novo Nordisk Foundation Center for Stem Cell Research, Faculty of Health and Medical Sciences, University of Copenhagen, Copenhagen, Denmark

**Keywords:** CEACAM5, CEA, immunohistochemistry, breast cancer, invasion

## Abstract

CEACAM5 is overexpressed in many primary breast carcinomas. However, the exact role of CEACAM5 in breast cancer tumorigenesis remains unresolved. Here, we examined a repository of 110 cryopreserved primary breast carcinomas by immunohistochemistry to assess the distribution of CEACAM5 in tumor subtypes. The majority of estrogen receptor-positive and HER2-overexpressing tumors were CEACAM5-positive, whereas most of Triple-negative tumors were negative. Assessing sample sets of paired primary breast cancers and corresponding lymph node lesions from a total of 59 patients revealed a high correlation between primary tumor and lymph node with regard to CEACAM5-status. However, a notable subset of sample sets demonstrated intratumoral heterogeneity in the primary tumor, the metastatic lesion or both, suggesting that both CEACAM5-positive and –negative cells can play a role in tumor dissemination. When examining the consequence of expression of CEACAM5 in breast cancer cell lines in culture assays we found that CEACAM5-expressing cells were less invasive. In survival analysis, using cohort studies of breast cancer, expression of CEACAM5 predicted different clinical outcomes depending on molecular subtypes. Altogether, our analysis suggests that CEACAM5 plays a context-dependent role in breast cancer that warrants further investigation.

## INTRODUCTION

The carcinoembryonic antigen family (CEA) consists of a subgroup of 12 members of carcinoembryonic antigen-related cell adhesion molecules (CEACAMs), and several of these are reportedly overexpressed in various cancers [1, 2]. CEACAM5, originally named Carcinoembryonic antigen (CEA), was first identified as a cancer-associated antigen in colon cancer [3]. Early work suggested that CEACAM5 was also often overexpressed in breast cancer [4]. Since then several immunobased assays have been implemented to examine the role of CEACAM5 as a clinically relevant marker in breast cancer. Generally, measuring CEACAM5-levels in serum is not considered for primary diagnosis in breast cancer, in large part because only a minor subset of patients has elevated levels [5, 6]. While some studies have demonstrated that increased serum levels in preoperative breast cancer patients do correlate to a worse outcome [5–8] others have not [9–11]. Similarly, work focusing on immunohistochemical analysis of breast cancer tissue have provided diverging conclusions. Work by Shousha and colleagues showed that expression of CEACAM5 in primary carcinomas correlated with lymph node metastases and with lower patient survival rates [12, 13]. While several reports essentially support these observations [14–16], a number of studies have failed to arrive at a similar conclusion [17–21]. To add to the complexity some noted a statistical significance only for subsets of the patient samples that was analyzed [22, 23] and one study even found an inverse correlation between CEACAM5-positivity and outcome [24]. Moreover, reports on the proportion of CEACAM5-positive breast cancers vary greatly, from a few to more than 80%, which at least in part may explain the discrepancies with regard to the clinical value of this marker [4, 13, 17, 21, 23, 25–27]. Finally, while most studies that noted the presence of intratumoral heterogeneity did not assess this further [13, 14, 19, 21, 22], one did suggest that poorly differentiated tumors generally contained fewer positive cells [17]. A summary of the observed results are available in Table 1. Overall, the available data do not provide a consensus on the role of CEACAM5 in breast cancer.

**Table 1:** A selection of studies that analyzed CEACAM5 in breast cancer patients

Study	Experimental analysis	Results
Wu, et al. (2014) [5]	Measurement of preoperative serum marker levels, correlated to patient outcome	• CEA levels were increased in 7.2% of patients • Overall prognosis was worse in patients with increased serum CEA levels
Shao, et al. (2015) [6]	Measurement of preoperative serum marker levels, correlated to patient outcome	• CEA levels were increased in 10.9% of patients • Elevated preoperative CEA serum levels was an independent prognostic marker for breast cancer
Ebeling, et al. (2002) [7]	Measurement of pre- and postoperative serum marker levels, correlated to outcome	• Elevated preoperative CEA serum levels were correlated to death from disease • A significant drop in postoperative CEA serum levels was correlated to death from disease
Imamura, et al. (2018) [8]	Measurement of pre- and postoperative serum marker levels, correlated to outcome	• Disease-free survival was worse in patients with high CEA serum levels
Given, et al. (2000) [9]	Measurement of pre- and postoperative serum marker levels, correlated to predictive value for recurrence	• CEA was a poor predictor of recurrence
Arslan, et al. (2000) [10]	Measurement of preoperative serum marker levels, correlated to tumor size and axillary lymph node invasion	• No significant correlation between baseline serum levels of CEA and tumor size/lymph node invasion
Svobodova, et al. (2018) [11]	Measurement of postoperative serum marker levels, correlated to disease recurrence	• No significant correlation between postoperative CEA serum levels and recurrence
Shousha, et al. (1978) [12]	IHC on primary breast tumors and corresponding lymph node metastases	• Significant relationship between CEA in primary tumors and presence of lymph node metastases
Shousha, et al. (1979) [13]	IHC on breast carcinomas, correlated to patient survival rates	• Patients with CEA-positive tumors had lower 5- and 10-year survival rates • 80% of tumors were positive • Most tumor cells stained in all positive cases
Mansour, et al. (1983) [14]	Measurement of preoperative serum marker levels and IHC on patients with stage I or II breast cancer, correlated to recurrence	• Patients with CEA-positive tumors had higher recurrence rate • Intensity and proportion of positive cells varied in each case
Saadatmand, et al. (2013) [15]	IHC on primary breast tumors, correlated to patient relapse	• Above median expression of CEA resulted in shorter relapse-free period
Liu, et al. (2019) [16]	IHC on primary breast tumors, correlated to malignant clinical features	• Elevated CEA expression was related to poor prognosis
Walker (1980) [17]	IHC on breast tumors, correlated to recurrence	• CEA-positivity correlated to good histological differentiation, but not to recurrence • 50% of carcinomas were positive • In poorly differentiated tumors with CEA, small numbers of positive cells were present
Gilchrist, et al. (1985) [18]	IHC on breast tumors, correlated to disease-free interval	• No association between CEA-positivity and biological course of the cancers
Robertson, et al. (1989) [19]	IHC on primary breast carcinomas, correlated to clinical parameres	• No correlation between CEA-positivity and lymph node stage, locoregional recurrence, disease-free interval or patient survival • The distribution of staining varied between tumors and within individual tumors
Eskelinen, et al. (1993) [20]	IHC on primary breast carcinomas, correlated to axillary lymph node status	• CEA-positivity was not correlated to axillary lymph node status
Mauri, et al. (1998) [21]	IHC on infiltrating breast carcinomas, correlated to clinical outcome	• CEA immunoreactivity was not prognostically relevant • CEA-reactive cells were seen in 45.2% of cases • The percentage of reactive cells ranged from 0% to 95% of tumor cells
Kuhajda, et al. (1983) [22]	IHC on primary breast tumors and axillary lymph nodes	• A trend for infiltrating ductal carcinomas with strong CEA staining to associate with synchronous axillary lymph node metastasis • Overall, CEA was present in 68% of cases • Heterogeneity was a notable feature in all *in situ* and infiltrating carcinomas
Esteban, et al. (1994) [23]	IHC on stage I and II breast carcinomas, correlated to histologic and clinical parameters	• CEA was an independent predictor of disease-free survival and overall survival in ER-negative patients • 56% of carcinomas were CEA-positive
Sundblad, et al. (1995) [24]	IHC on stage I and II breast carcinomas correlated to clinical parameters	• An association between absence of CEA-staining and recurrence of disease was observed
Goldenberg, et al. (1978) [25]	IHC on primary breast carcinomas	• CEA staining was demonstratable in 1.6% of breast carcinomas
Croce, et al. (1997) [26]	IHC on primary breast cancers	• CEA expression was present in a small proportion of breast tumors
Blumenthal, et al. (2007) [27]	IHC on primary breast cancers	• Expression of CEA were significantly lower than CEACAM6, generally comparable to background levels
Heyderman, et al. (1977) [4]	IHC on primary breast carcinomas	• CEA staining was present in 10/12 (83%) of breast carcinomas

Here, we assess CEACAM5 expression in breast cancer subtypes by immunohistochemistry, and compare the expression pattern in primary tumors to corresponding lymph node metastases. Based on these results as well as experimental data performed on CEACAM5-expressing cell lines, we devise a hypothesis of a subtype-dependent role of CEACAM5 in breast tumor dissemination.

## RESULTS

### Antibodies CB30 and COL-1 specifically target CEACAM5-positive breast cells

**Figure 1:**
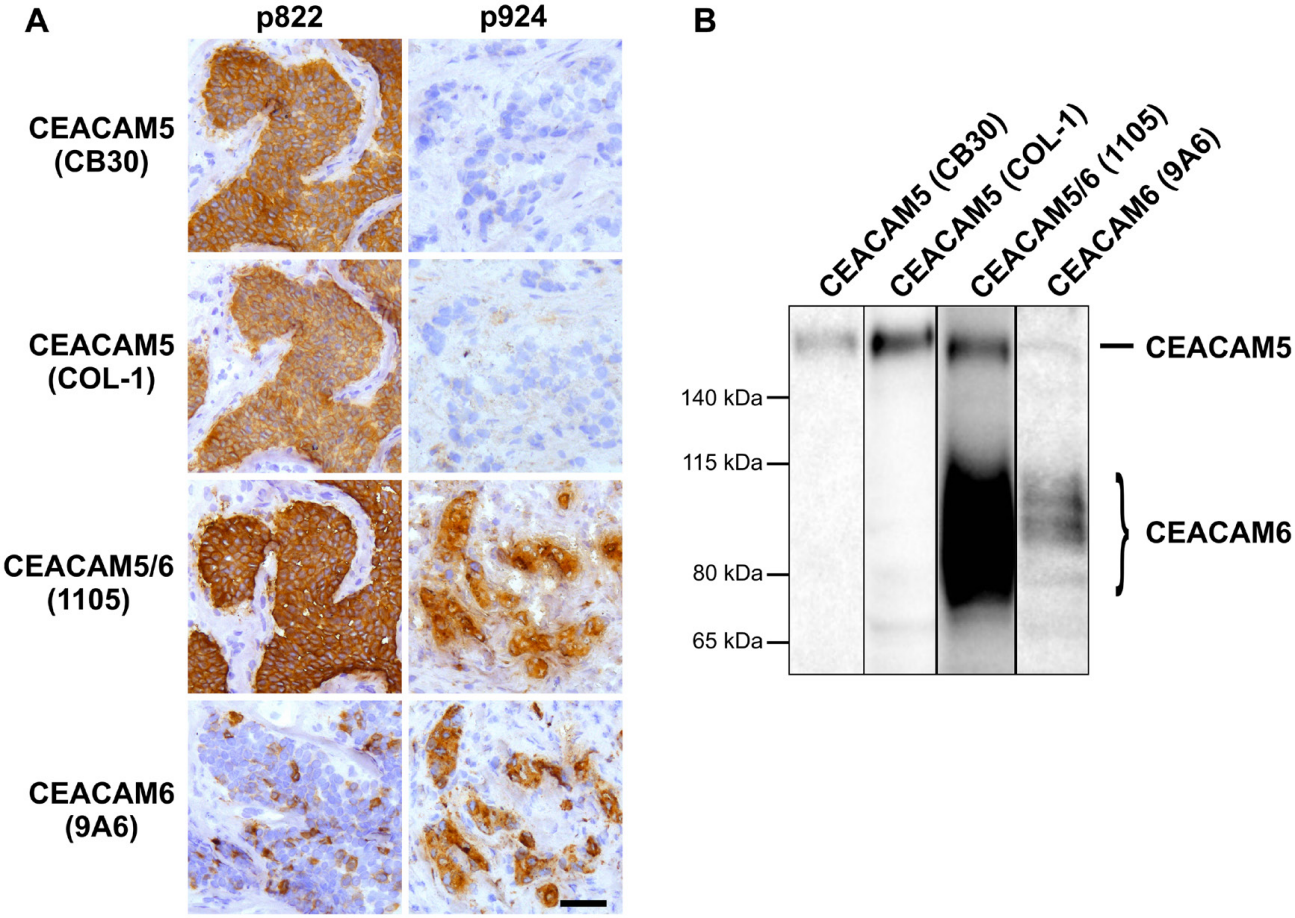
Antibodies CB30 and COL-1 specifically target CEACAM5-positive cells. (**A**) Immunohistochemical staining of two breast tumors with mAbs: CB30, COL-1, 1105 and 9A6. Bar, 50 μm. (**B**) Western blot performed on MCF7i cells demonstrating that mAb 1105 cross-reacts with CEACAM6 while mAbs CB30 and COL-1 are highly specific for CEACAM5.

In a search for CEACAM5-specific antibodies that work optimally for immunohistochemistry on cryosectioned breast cancer tissue, we tested three monoclonal antibodies (mAbs): CB30, COL-1 and 1105, all of which have been utilized in previous studies by other groups [28–30]. Apart from an occasional stromal background reaction, the staining pattern of mAb COL-1 was essentially the same as mAb CB30 in breast carcinomas (Figure 1A). However, mAb 1105 showed much more reactivity compared to mAbs CB30 and COL-1. Particularly, we observed a clear discrepancy in some tumor samples where positive staining was evident with mAb 1105, while completely negative with mAbs CB30 and COL-1 (Figure 1A and Supplementary Table 1). In these biopsies, we found that the staining pattern of mAb 1105 was correlated with expression of another CEA family member, CEACAM6 (Figure 1A). In a previous study, we have evaluated CEACAM6-expression in breast carcinomas [31]. To corroborate these immunohistochemical staining results, we performed western blotting on breast cancer cell extracts, and confirmed the specificity of CB30 and COL-1 for CEACAM5 as well as the cross-reactivity of 1105 with CEACAM6 (Figure 1B). Furthermore, RT-PCR analysis on breast carcinomas confirmed the specificity of CB30 to CEACAM5 (Supplementary Figure 1A and 1B). Finally, to ensure that any staining could not be accredited to cross-reaction to the widely expressed CEACAM family member, CEACAM1 [1] we confirmed that CB30 and COL-1 did not recognize any epitopes on CEACAM1 (Supplementary Figure 1C).

In order to determine if expression of CEACAM5 can be accredited to a differentiation hierarchy in a manner similar to CEACAM6 [31], we examined the distribution of CEACAM5 in normal breast tissue. However, quantification was not possible due to the apical distribution of the antigen and scarcity of positive cells, as reaction was confined to lobular acini with low frequency (Supplementary Figure 2A). As an alternative method, we utilized immunosmear-stainings on trypsinized cells from normal breast tissue (*n* = 4) which revealed that CEACAM5-positive cells constituted an average of 1.6 ± 1.9% of luminal cells and that they were mainly part of a mature luminal compartment (Supplementary Figure 2B and 2C). These data highlight the importance of utilizing specific CEACAM5 antibodies for analysis of breast tissue and demonstrate that the minor subset of CEACAM5-positive cells present in the normal breast gland preferably belongs to a differentiated luminal compartment. In our hands mAbs CB30 and COL-1 were antibodies that performed reliably in immunohistochemistry on normal and malignant breast tissue.

### The majority of estrogen receptor-positive and HER2-enriched breast carcinomas are CEACAM5-positive, while most Triple-negative tumors are negative

**Figure 2:**
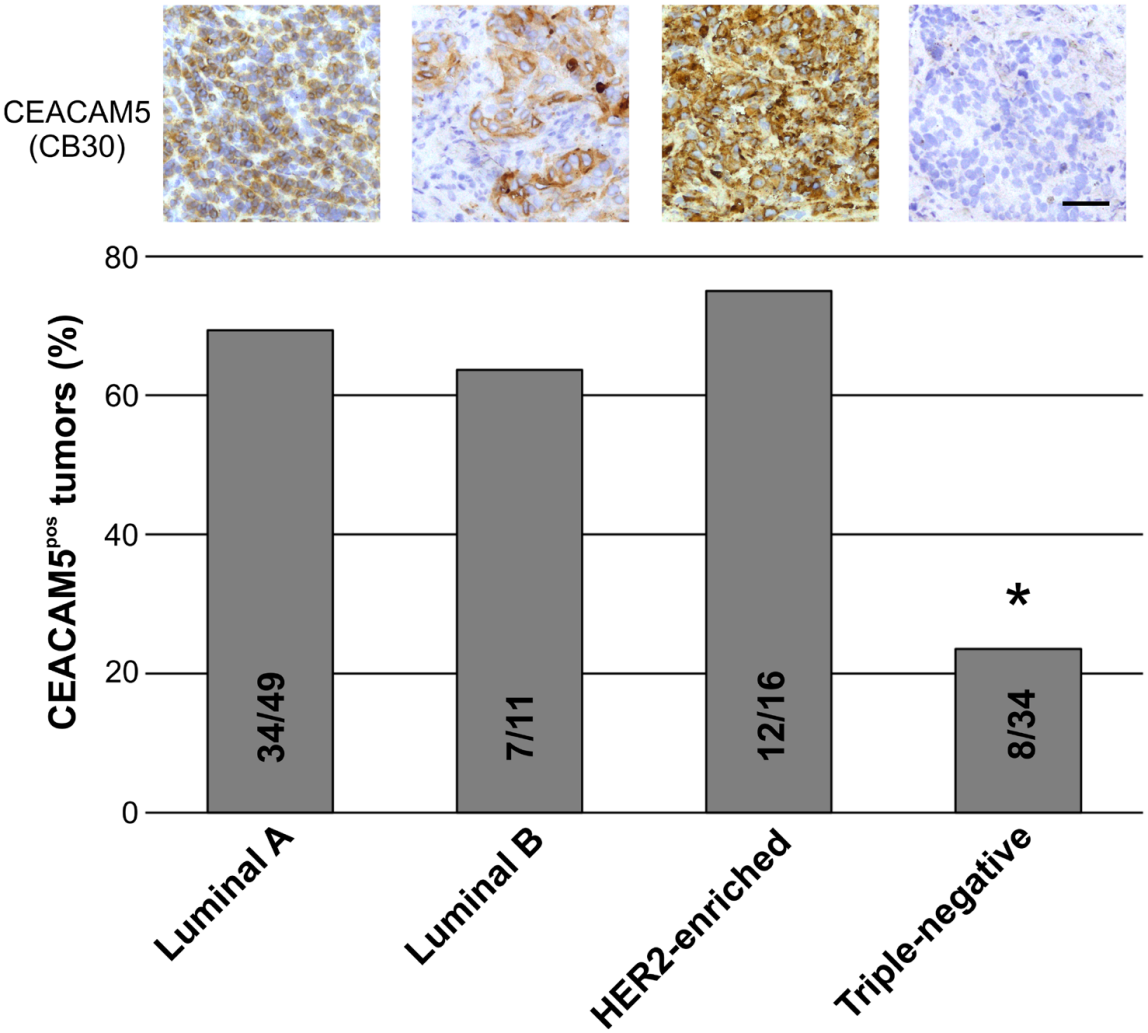
The proportion of CEACAM5-positive tumors are significantly higher in non-TN breast cancer subtypes. A bar graph showing the proportion of Luminal A, Luminal B, HER2-enriched and Triple-negative (TN) breast carcinomas positive for CEACAM5. ^*^indicates *p* < 0.05 tested by ANOVA with Tukey’s significance test. Representative immunostained tumors are shown in top panel. Bar, 50 μm.

To analyze the distribution of CEACAM5 expression in different subtypes of breast tumors, we examined a sample of frozen biopsies of which some previously have been utilized for testing CEACAM6-expression [31]. A total of 110 breast tumors were subjected to immunohistochemical assessment by use of CEACAM5-specific mAbs CB30 and COL-1, CEACAM6 cross-reacting 1105 and CEACAM6-antibody 9A6. Here, tumors with > 1% of the neoplastic cells expressing CEACAM5 were considered positive. For CEACAM5-positive tumors a wide range of intensity and frequency was observed in the cytoplasm and membrane of neoplastic cells. There was overall agreement between CB30 and COL-1 for the tumor assessment (Supplementary Table 1). Importantly, we found that the distribution of CEACAM5 expression significantly differed among breast cancer subtypes. Of the 49 breast carcinomas that were among the Luminal A subtype, 34 (69%) carcinomas were CEACAM5-positive (Figure 2). The majority of Luminal B (7 out of 11 carcinomas, 63%) and HER2-enriched (12 out of 16, 75%) tumors were also positive for CEACAM5. However, of the 34 Triple-negative (TN) tumors only 8 (24%) expressed CEACAM5. A similar subtype-dependent pattern was observed with CEACAM6 staining (Supplementary Table 1). The majority of CEACAM5-positive tumors concomitantly expressed CEACAM6 with few exceptions, as CEACAM6 (76 out of 110, 69% positive) was more widely expressed than CEACAM5 (61 out of 110, 55% positive) (Supplementary Table 1).

These data demonstrate that expression of CEACAM5 is correlated with breast tumor subtypes, as the most differentiated subtypes are positive for CEACAM5, while TN tumors, which are less differentiated [32], are mostly negative for CEACAM5.

### Comparison of CEACAM5 expression in invasive primary tumors and corresponding lymph node metastases

**Figure 3:**
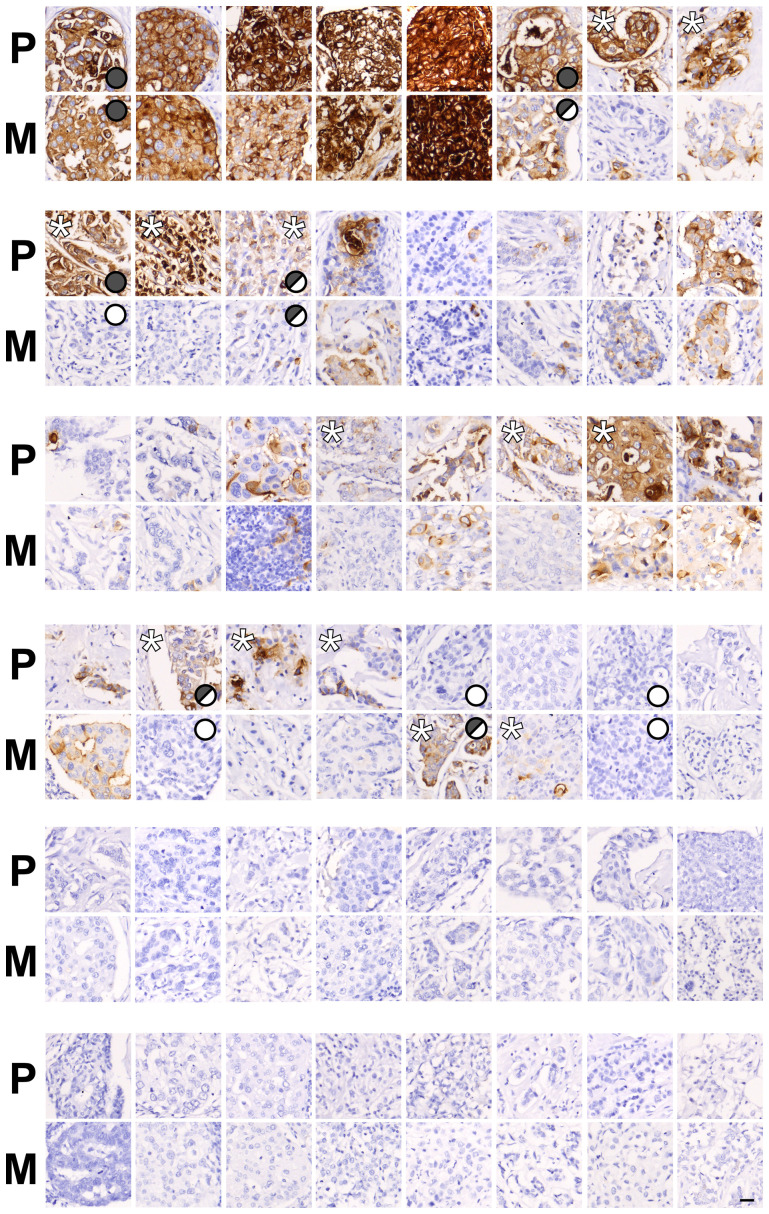
Pairs of primary breast cancer and lymph node metastases demonstrate different patterns of CEACAM5 expression. Immunohistochemical stainings for CEACAM5 on paired primary breast cancers (P) and corresponding lymph node metastases (M) from a tissue microarray (TMA). Tumor pairs are ordered according to expression patterns marked by symbols: homogeneous expression (filled grey circle), heterogeneous expression (half-filled grey circle) and no expression (white circle). ^*^marks tumors with a notably higher frequency of CEACAM5-positive cells within a pair. Bar, 25 μm.

Next, we questioned whether the expression pattern of CEACAM5 in invasive primary tumors correlates with the pattern found in paired lymph node metastases. To test this, we first immunohistochemically examined CEACAM5 expression patterns of a small sample of 11 pairs of frozen invasive primary breast carcinomas with corresponding lymph node metastases using mAb CB30. In 10 of these tumor pairs there was accordance between the primary tumor and lymph node lesion, of which 3 were determined negative (Supplementary Figure 3). However, in one set, which is coincidently a male breast carcinoma, the lymph node lesion was negative for CEACAM5, while the primary tumor was heterogeneously positive. In order to substantiate these observations, we evaluated a tumor micro array (TMA) consisting of 50 pairs of paraffin-embedded metastatic primary breast carcinomas and corresponding lymph node metastases for expression of CEACAM5. Thus, we divided tumors into three categories: homogeneously positive, heterogeneously positive and negative. Of the 48 tissue cores that were evaluable, 28 (58%) primary tumors were CEACAM5-positive, either heterogeneously (18 out of 28) or homogeneously (10 out of 28) (Figure 3). For 20 of these tumors the expression pattern of CEACAM5 in the primary tumor was in the same category as the corresponding metastatic tumor. In three tumor pairs the primary tumor was homogeneously positive, while the metastatic lesion demonstrated heterogeneity. Five primary tumors that were either homogeneously or heterogeneously positive were negative when analyzing the metastasis while 2 tumor pairs demonstrated a negative primary tumor with a heterogeneously positive metastasis. The remaining 18 CEACAM5-negative primary tumors were also negative in the metastatic lesion.

Arguably, these data suggest a complex role of CEACAM5 in breast cancer and implies that both CEACAM5-positive and -negative cells can be directly involved in invasive and metastatic processes.

### CEACAM5-negative breast cancer cells are more invasive than CEACAM5-positive in culture

**Figure 4:**
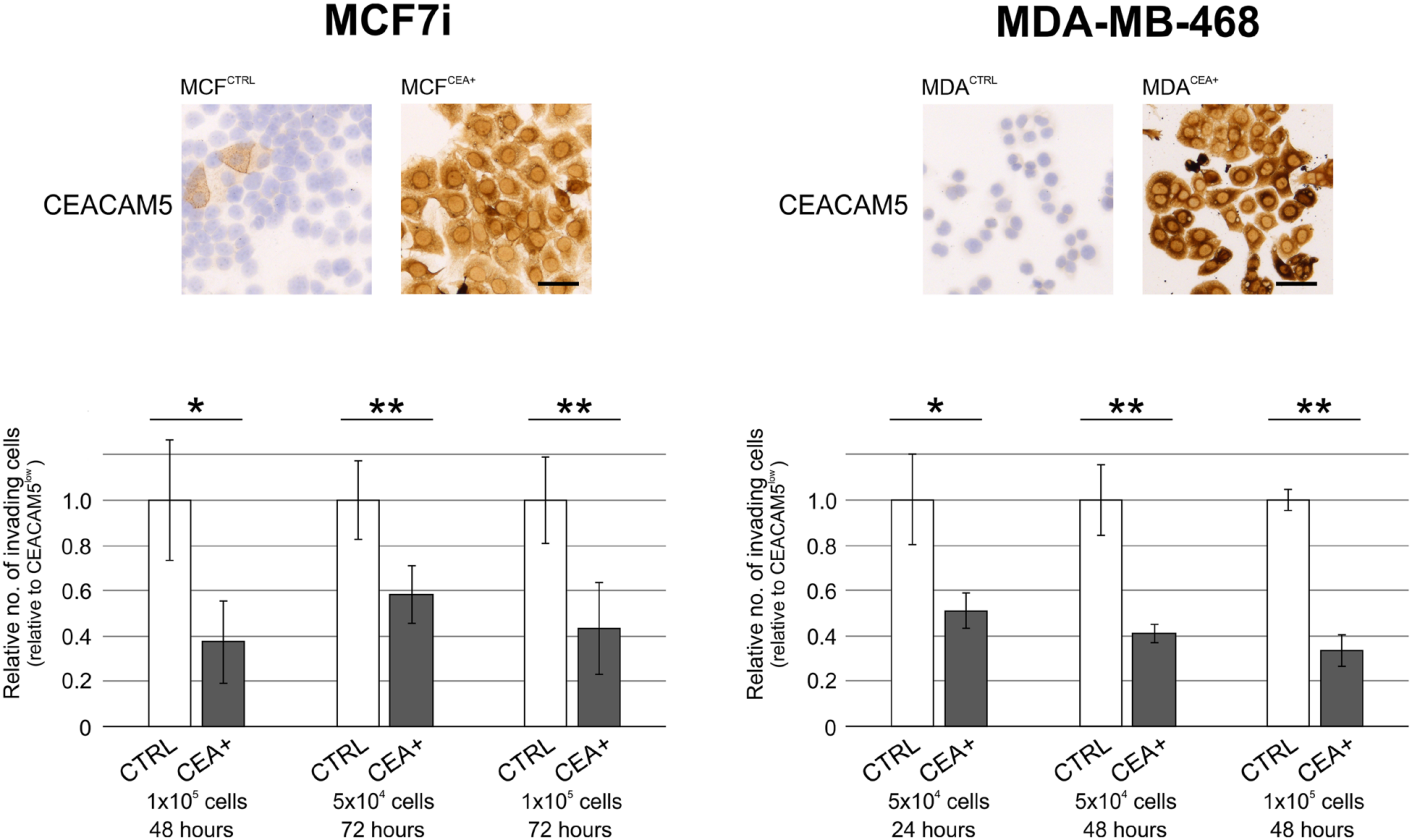
CEACAM5-positive breast cancer cells are less invasive in culture. Comparison of MCF7i and MDA-MB468 breast cancer control cells (CTRL) with ectopically CEACAM5-overexpressing sublines (CEA+) by immunostaining for CEACAM5 (top panels) and by invasion through Matrigel coated filters (lower panels). Each cell line was analyzed in three different conditions with regard to no. of cells on filter, and invasion time (listed under graphs). In graphs the relative invasion of CTRL cells were normalized to 1. ^*^ and ^**^ indicate *p* < 0.05 and *p* < 0.01 tested by *t* test, respectively. Bars, 50 μm.

To examine the role of CEACAM5 in the process of tumor dissemination of breast cancer cells, we measured the invasive capacity in a Matrigel-coated transwell filter invasion assay using two different breast cancer cell lines. When CEACAM5 was overexpressed, MCF7i and MDA-MB-468 breast cancer cells became less invasive than control cell lines where low endogenous levels of CEACAM5 were exhibited (Figure 4). Testing expression of CEACAM1 in the overexpressing cell lines demonstrated that this CEACAM family member was not co-induced with CEACAM5 (Supplementary Figure 4). Invasion data were substantiated by experiments on MCF7i involving cells with endogenous CEACAM5-expression. Here, CEACAM5^neg/low^ cells were generally more invasive than CEACAM5^high^ cells as determined by fluorescence immunocytochemistry or after sorting by flow cytometry (Supplementary Figure 5).

Thus, these results demonstrate that breast cancer cells expressing CEACAM5 retain a lower capacity of tumor-cell dissemination.

### The clinical significance of CEACAM5 expression varies with breast tumor subtype

**Figure 5:**
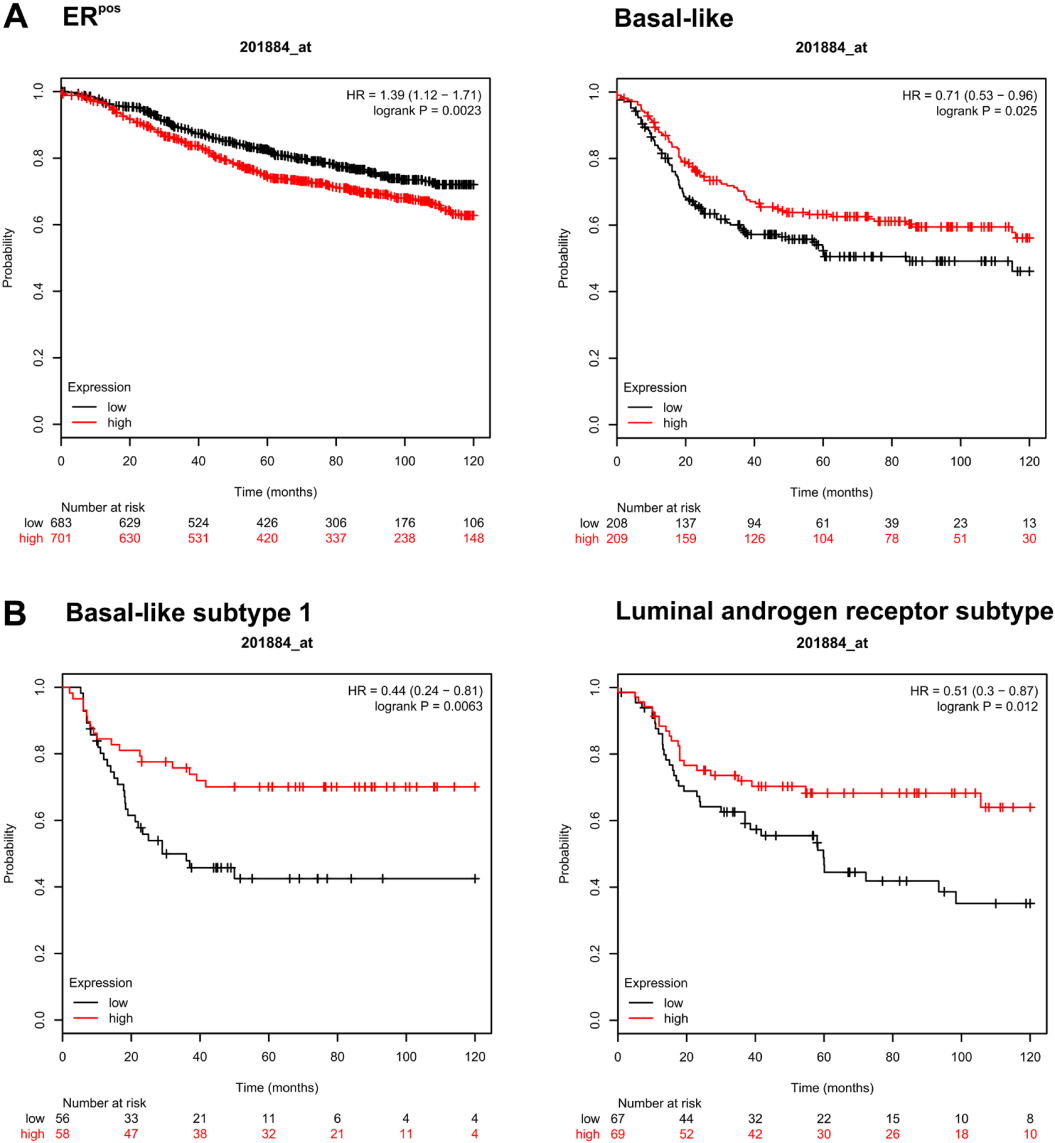
The clinical significance of CEACAM5 expression varies with breast tumor subtype. (**A**) Kaplan-Meier plots of subsets of breast cancer patients (ER-positive and Basal-like) analyzed for relapse-free survival when trichotomized into highest and lowest tertiles with regard to *CEACAM5* expression. (**B**) Similar analysis performed on two subsets of TN tumors (Basal-like subtype 1 and Luminal androgen receptor subtype). Data are also presented in Supplementary Table 2.

To get some insight to the clinical relevance of expression of CEACAM5 in breast cancer, we utilized an online-based tool to draw survival plots of patient groups divided by gene expression levels from breast cancer cohort studies [33]. Predicted prognostic effects of high and low expression of *CEACAM5* in different subtypes on clinical outcomes are summarized in Supplementary Table 2. Comparing patients trichotomized into *CEACAM5*^high^ and ^-low^ groups for relapse-free survival (RFS), clinical outcomes were different between subtypes of breast cancer. A High level of *CEACAM5*-expression was significantly unfavorable for patients with ER-positive tumors, whereas it was more favorable for patients with Basal-like tumors (Figure 5A). Furthermore, a more favorable prognostic effect of high levels of CEACAM5 was observed in some sub-clusters of the TN tumor subtype, including Basal-like 1 and Luminal androgen receptor subsets of tumors (Figure 5B).

In total, these data suggest a multifaceted role for CEACAM5 that depends on breast cancer subtype with regard to carcinogenesis.

## DISCUSSION

Immunohistochemical analysis is a useful tool to identify different types of breast cancers, which in turn provides valuable information on a feasible therapeutical strategy as well as overall prognostic consequences. However, routine analyses of breast cancers rely on relative few markers that include steroid hormone receptors and HER2. Efforts to expand on the current combination of reliable breast cancer markers require a thorough characterization of potential candidates – both regarding expression patterns and functional consequences. In this study, we initiated an investigation of CEACAM5, as there is still a lack of consensus on the consequence of CEACAM5-expression in breast carcinomas. In our sample of 110 breast carcinomas, 55% were CEACAM5-positive by immunohistochemistry which is in line with several of the previous reports [21–24]. However, depending on studies, frequencies have ranged from less than 2% to more than 80% [4, 13, 20, 25, 26]. In part, the discrepancies may be explained by differences in the composition of the sample material, as we observed that TN tumors are more often CEACAM5-negative than ER-positive and HER2-enriched tumors. Moreover, concerns on the lack of specificity for some antibodies has also been expressed [22]. We found that mAb 1105 reacts with CEACAM6 as well as CEACAM5, whereas mAbs of CB30 and COL-1 share the high specificity and sensitivity for CEACAM5. Cross-reacting CEACAM5-antibodies, including mAb 1105, are still commercially available, which means that experiments where they have been utilized should be evaluated with caution [15, 30, 34].

As mentioned above, among breast cancer subtypes, the frequency of CEACAM5-positivity was notably lower in TN carcinomas (24%) compared to other subtypes (> 60%). In earlier work, Dr. Walker did note that poorly differentiated grade III carcinomas were generally less positive than more differentiated grade I and grade II tumors [17]. This is in line with our observation as the majority of TN breast carcinomas are generally considered to be grade III [32]. Furthermore, based on preoperative serum levels, two studies have shown that a lower proportion of women with TN breast cancer have elevated CEACAM5 compared to other molecular subtypes [5, 35]. Collectively, these data indicate that expression of CEACAM5 could relate to a differentiation hierarchy. In normal breast, we demonstrated that in fact a minor subset of normal luminal breast epithelial cells do express CEACAM5, and that positive cells preferably are present as part of a mature luminal compartment. While it has not been reported previously by immunohistochemistry [17, 22, 23, 29], presence of *CEACAM5* in normal breast tissue has been detected with RT-PCR [36]. Previously, we have examined CEACAM6 and found a similar correlation to a differentiation hierarchy both in normal epithelial tissue and in breast cancer [31]. Thus, it is highly plausible that CEACAM5 is a marker of epithelial differentiation – in normal breast and in corresponding cancer.

When analyzing TMAs with relatively small cores using markers that often show considerable intratumoral heterogeneity the results warrants a cautious interpretation. However, TMAs are routinely used as a resource for screening in clinical oncology [37]. After comparing expression patterns in sets of primary breast tumors with corresponding lymph node metastases using commercially available TMAs, we hypothesized that CEACAM5-negative cancer cells may participate in tumor dissemination. This was supported by experimental data showing that CEACAM5-overexpressing breast cancer cells were less invasive. Even if CEACAM5-positive cells are less invasive *per se* this does not leave out a role for CEACAM5 with regard to other processes of tumor dissemination when considering formation of metastases. Recently it was demonstrated that non-small lung cancer cells could carry a sialyl Lewis x/a motif on CEACAM5 which potentially aids these cells in vascular extravasation [38]. Furthermore, the work of Powell and colleagues on a patient-derived xenograft (PDX) breast cancer model came to the conclusion that when establishing lung metastases tumor cells generally upregulated CEACAM5 in a process considered to be mesenchymal to epithelial transition (MET) [39]. Thus, at various stages during tumor dissemination some CEACAM5-competent cells may benefit from upregulating CEACAM5 by differentiating or undergoing MET.

**Figure 6:**
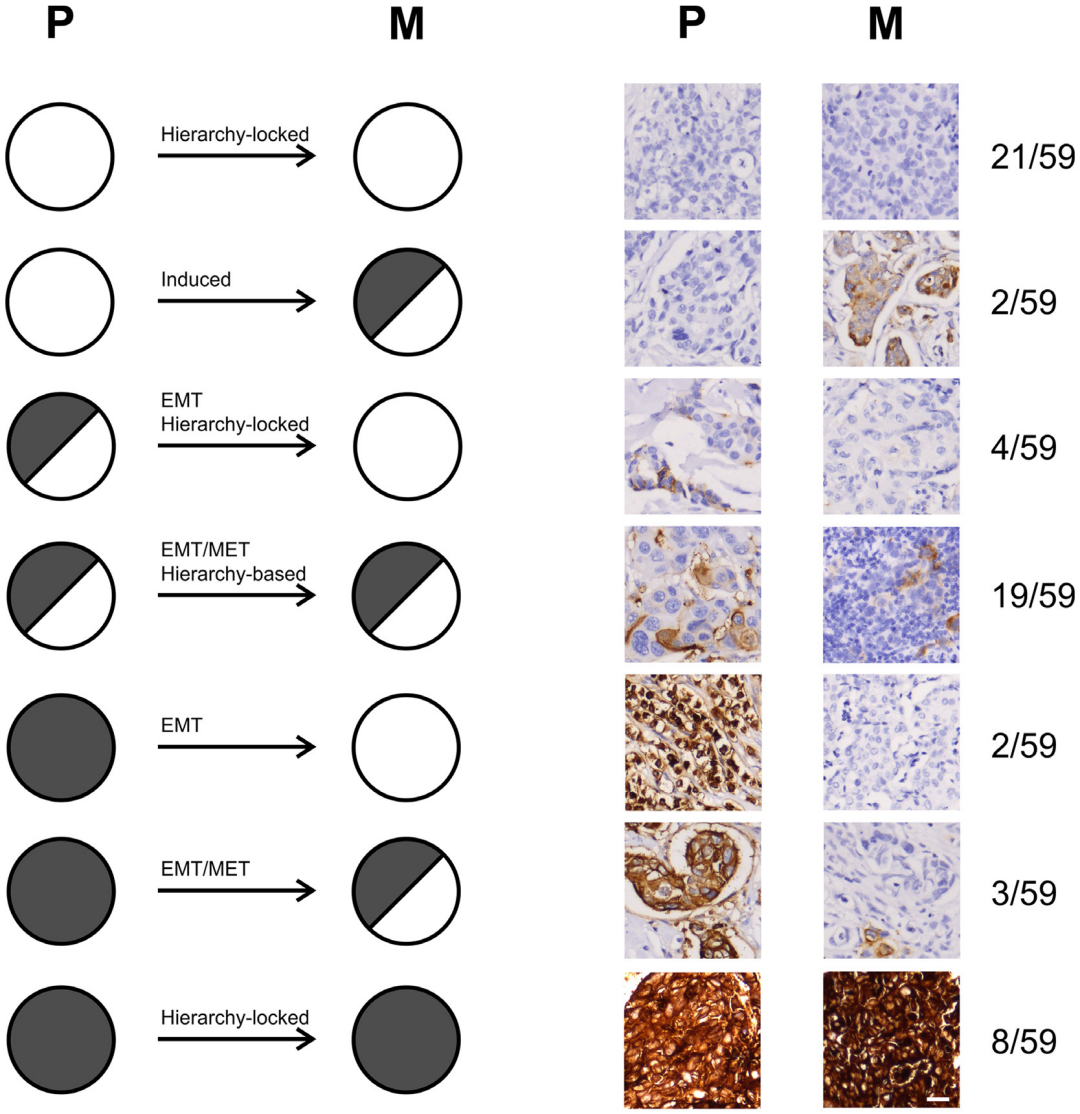
Proposed mechanisms of tumor dissemination from primary breast tumors to metastases. CEACAM5-expression outlined as no expression (white circle), heterogeneous expression (half-filled grey circle) and homogeneous expression (filled grey circle) based on the observed expression patterns in a total of 59 sets of primary breast carcinomas (P) and corresponding lymph node metastases (M), including datasets from both cryosectioned tissue and TMA. The distribution of the 59 tumor sets are outlined along with a representative immunostain at the right, as well as the proportion of tumor-sets with the given profile. *Hierarchy-locked* suggests that the disseminating tumor cells remain in a specific differentiation state. *Induced* suggests that extrinsic factors lead to induction of CEACAM5 in negative cells. *EMT* and *EMT/MET* suggest that disseminating tumor cells undergo epithelial to mesenchymal transition (EMT) without or with subsequent mesenchymal to epithelial transition (MET), respectively. *Hierarchy-based* suggests that disseminating cancer stem cells retain their differentiation capacity.

When taking into account that CEACAM5 expression in breast carcinomas may be a marker both for epithelial differentiation and for transition between epithelial and mesenchymal-like states we propose that different mechanisms are in effect during tumor cell dissemination dependent on the composition and hierarchical distribution of cells in the primary tumor (Figure 6).

Analysis of clinical data using a publically available webtool [33] comparing expression of mRNA transcripts in tumors of breast cancer patients revealed interesting differences that depend on tumor subtype. While high *CEACAM5* was correlated with shorter RFS for patients with ER-positive tumors, high *CEACAM5* increased RFS in patients with subsets of Basal-like tumors. This clearly demonstrates that the implications of CEACAM5-expression in tumors are complex. The importance of CEACAM5 in tumor cells disseminating to lymph nodes and to more distant sites like lung tissue may even differ [39, 40]. As has been found for other markers, a notable discordance in expression of CEACAM5 between primary tumor and metastasis may have implications for choosing treatment strategies after surgical removal of the primary tumor [41].

Moreover, the fact that 93% of CEACAM5-positive breast carcinomas in this study also express CEACAM6 suggests that multiple CEACAMs may act in concert, further complicating the consequences of CEACAM5-expression.

Overall, the findings in this study may help improve the understanding of the biological effect of CEACAM5-expression in breast cancer.

## MATERIALS AND METHODS

### Breast tissue

Breast cancer samples were obtained anonymously from breast cancer patients operated at Rigshospitalet, Region H, Copenhagen, Denmark. The storage and use of human material has been reviewed and approved by the Regional Scientific Ethical Committees (H-2-2011-052 and H-3-2010-095) and the Danish Data Protection Agency (2011-41-6722) and has been handled according to established guidelines in subsequent experiments. Normal breast tissue was obtained from women undergoing reduction mammoplasty for cosmetic reasons at Capio CFR Hospitaler, Hellerup or Lyngby, Denmark. The personal information of the donors was kept confidential and protected. Subsequent handling of normal tissue for cryopreservation or single cell dissociation was done as previously described [42]. A paraffin-embedded tumor micro array (TMA) with tissue cores of primary tumors and corresponding lymph node metastases from 50 breast cancer patients was purchased a commercial provider (BR1005b, US Biomax).

### Immunostainings

Cryosections (6 μm) of human breast tissue were fixed with either methanol for 5 minutes minutes at –20°C or 3.7% formaldehyde in phosphate buffered saline (PBS), pH 7.4, for 10 minutes at room temperature. Formalin fixed sections were then rinsed two times with PBS and permeabilized with 0.1% Triton X-100 for 10 minutes. Both formalin and methanol fixed sections were incubated in Ultra V Blocking buffer (Thermo Scientific) for 5 minutes following incubation of primary antibodies diluted in 10% goat serum in PBS as follows: CEACAM5 (CB30, 1:100, Thermo Fisher,) CEACAM5 (COL-1, 1:100, Thermo Fisher), CEACAM5 (1105,1:100, Thermo Fisher), CEACAM6 (9A6, 1:200, Abcam), EpCam (9C4, 1:50, Biolegend), ERα (1D5, 1:100, Dako), PR (PgR636, 1:100, Dako), HER2 (TAB250, 1:100, Invitrogen), for 60 minutes at room temperature. Sections were then washed 3 times with PBS, left with PBS for 5 minutes and washed 3 times again prior incubation with Ultravision ONE HRP Polymer (Thermo Scientific) for 30 minutes at room temperature. Finally, samples were washed with PBS as before and visualized by incubating with 3.3′-Diaminobenzidine solution (DAB, Sigma) activated with 0.02% hydrogen peroxidase (Merck). Nuclei were counterstained by haematoxylin (Sigma). A similar procedure was followed for staining cell cultures and paraffin-embedded TMA (BR1005b, US Biomax). Pre-treatment of the TMA included baking for 60 minutes at 60°C, deparaffinization and boiling for 10 mins in TEG buffer, pH9. Cryostained breast cancers were divided into Luminal A (ERα^pos^ and/or PR^pos^ with no HER2-overexpression), Luminal B (ERα^pos^ and/or PR^pos^ and HER2-overexpressing), HER2-enriched (ERα^neg^ and PR^neg^ and HER2-overexpressing) and Triple-negative (ERα^neg^ and PR^neg^ with no HER2-overexpression). Tumors were generally considered positive when ≥ 1% of the neoplastic cells expressed the examined marker. For immunofluorescent staining of single cell smears and filters from invasion assays, after fixation cells were incubated with primary antibody; CEACAM5 (CB30, 1:50), CAM5.2 (CAM5.2 1: 25, BD Biosciences), CD117 (K45, 1:50, Thermo Fisher), Ks20.8 (Ks20.8, 1:10, Dako) for 2 hours followed by incubation with isotype-specific secondary antibody Alexa Flour 488 or Alexa Flour 568 (1:500, Invitrogen) for 30 minutes. Images were acquired on a Leica DM5500B microscope equipped with a DFC550 camera.

### Western blotting

Western blotting was performed as previously described [43]. In short, protein of breast cancer cell line MCF7i extracted by using RIPA lysis buffer were separated using electrophoresis of pre-casted 4–12% Novex™ Bis-Tris polyacrylamide gradient gels (Life technologies), which were then transferred onto a PVDF membrane overnight at 0.33A. For testing of cross-reactivity to CEACAM1 cell lysate from human embryonic kidney HEK293 overexpressing human CEACAM1 was utilized (Sino Biological Inc.). Primary antibodies CEACAM5 (CB30, 1:1000) CEACAM 5 (COL-1, 1:1000), CEACAM5 (1105, 1:1000), CEACAM6 (9A6, 1:1000), CEACAM1 (B3-17, 1:1000, Millipore) or β actin (AC-15, 1:5000, Sigma) were diluted in TBS tween buffer with 5% BSA and secondary antibody conjugated with horseradish peroxidase (HRP) (1:2000, DAKO, P0447) were diluted in 5% milk-TBST. Chemiluminescent detection method using ECL solutions (Pierce ECL, Thermo Scientific) and photographic images were captured in a chemiluminescence imager, Amersham Image 600 (GE Healthcare Life Sciences).

### Fluorescence-Activated Cell Sorting (FACS)

FACS sorting was used to obtain CEACAM5^high^ and CEACAM5^neg/low^ from breast cancer cell lines in culture. For this procedure, cells were dissociated into single cells using Accutase (Milipore), spun down and resuspended in Hepes buffer. Primary antibody was added directly to the cell plus buffer solution: CEACAM5 (CB30, 1:50 or COL-1, 1:100), and incubated for 30 minutes at 4°C. Cells were washed two times with Hepes buffer and incubated with isotype-specific secondary antibody Alexa Flour 488 or Alexa Flour 647 (1:500, Invitrogen) for 20 minutes at 4°C. Cells were washed as before and passed through a 20 μm filter cup (Falcons). Fixable viability stain 780 (1:1000, BD Biosciences) were used to distinguish living from dead cells. Analysis and sorting was performed on either a FACSAria II or FACSFusion instrument (BD Biosciences). Around 100.000 sorted cells were seeded in T25 flasks for further experiments. The experiments that was performed with single-cell sorting were not included in this version of the manuscript. Luminal cells for immunosmear stainings were sorted from uncultured primary cells utilizing p75 and EpCAM as described previously [44].

### Cell culture

The breast cancer cell line MCF7 was utilized to generate a more invasive subline, MCF7i, by harvesting and expanding cells that invaded through Matrigel-coated FluoroBlok filters (Corning), as described under Invasion Assay. MCF7i and parental MCF7 were cultivated in DMEM 1965 medium supplemented with 2 mmol/L glutamine, 7 nonessential amino acids solution (Gibco), 6 ng/L insulin (Sigma), 50 μg/ml gentamycin (Biological Industries) and 5% FBS. MDA-MB-468 breast cancer cells were cultured in DMEM-F12 medium supplemented with 2 mmol/L glutamine, 50 μg/ml gentamycin and 10% FBS.

### Plasmids and viral transduction

Human CEACAM5 gene in pLV-C-GFPSpark tag lentiviral plasmid and control plasmid were purchased from Sino Biological (HG11077-ACGLN). For lentiviral production, HEK 293FT cells were transfected with pCMV-Δ.8.91 and pCMV-VGVg plasmids via the calcium phosphate transfection method. Viral supernatants were collected 48 hours post transfection and filtered through a 0.45 μm filter prior to transduction. Subsequently, transduced cells were selected by FACS using a combination of expression of GFP and CEACAM5.

### Invasion assay

Prior to the assay, cultured cells were starved in DMEM/F12 medium with 1% serum for 24 hours. Cells were then detached and separated into single cells using Accutase (Milipore). A total number of 5 × 10^4^ cells were seeded in 1% FBS onto Matrigel-coated 24-well FluoroBlok™ inserts with 8 μm pores (Corning) in quadruplicates, and inserts were then transferred onto 10% FBS DMEM/F12 medium for 24–72 hours. For MCF7i cells sorted into CEACAM5^high^ and CEACAM5^neg/low^ fractions cells were acclimatized in normal growth medium for at least 48 hours before medium-starvation. After fixation in ice cold methanol, filters were cut from the inserts and mounted on a glass slide with DAPI containing mounting medium (Invitrogen). Images were acquired by fluorescence microscopy at 20× from 6 to 9 randomly picked areas, and nuclei from invaded cells were counted manually. In one experiment immunofluorescence staining was performed on the filters before mounting, and images were acquired from both sides of the filter.

### RNA extraction and real time - qPCR

Cryosectioned breast carcinomas were dissolved in Trizol (Invitrogen) by vortexing and and manually homogenized using a VWR™ Disposable Pestle (Argos Technologies). After running through QIAshredder™ columns (Qiagen), total RNA were extracted using the Direct-zol™ RNA miniprep protocol (Zymo research) and were reversely transcribed using the High Capacity RNA-to-cDNA Kit (Applied Biosystems). Quantified real-time PCR was performed using Taqman gene expression assays (Applied Biosystems) for CEACAM6 or SsoFast™ EvaGreen^®^ Supermix (Bio-Rad) for CEACAM5 and EpCAM on Bio-Rad CFX manager 3.0 and thermocycler (Bio-Rad). The primers used were: CEACAM5 (forward TTTCTCCCTATGTGGTCGCTCCAG, reverse AGCAG ATTTTTATTGAACTTGTGC) that were adapted from a web-based primer bank, EpCam (forward AGTGTACTTCAGTTGGTGCACAAA, reverse AGTGTCCTTGTCTGTTCTTCTGAC), and GAPDH (forward ACCACAGTCCATGCCATCAC, reverse TTCACCACCCTGTTGCTGTA), CEACAM6-FAM (Hs03645554_m1) GAPDH-VIC (Hs02758991_g1).

Gene expression was calculated by the 2^−ΔΔCt^ method.

### Kaplan Meier plots

KM-plotter (https://kmplot.com) which is a publically available web tool, was utilized to generate Kaplan-Meier plots based on datasets from > 6000 breast cancer patients [45]. Analysis of expression of *CEACAM5* in the datasets was performed using the JetSet best probe set (Affymetrix ID: 201884_at). Expression data were trichotomized into upper and lower tertiles to compare the patients with the highest levels versus the lowest levels of *CEACAM5* expression for relapse-free survival and overall survival. Basal-like subtype 1 and Luminal androgen receptor subtypes are subdivisions of TN breast cancer. The number of patients analyzed can be found in Supplementary Tables 2 and 3.

### Statistics

Estimated *p* values were calculated with a statistical programming language R (version 3.6.3) and its integrated development environment, R studio (version 1.2.5033).

## SUPPLEMENTARY MATERIALS


