# Supplementary material for: Heterogeneity of CEACAM5 in breast cancer
Source: Oncotarget. 2020 Oct 27;11(43):3886–99. doi: 10.18632/oncotarget.27778 (PMC7597409; doi:10.18632/oncotarget.27778)
Supplement: Supplementary file 1 [file oncotarget-11-3886-s001.pdf]

Heterogeneity of CEACAM5 in breast cancer

SUPPLEMENTARY MATERIALS

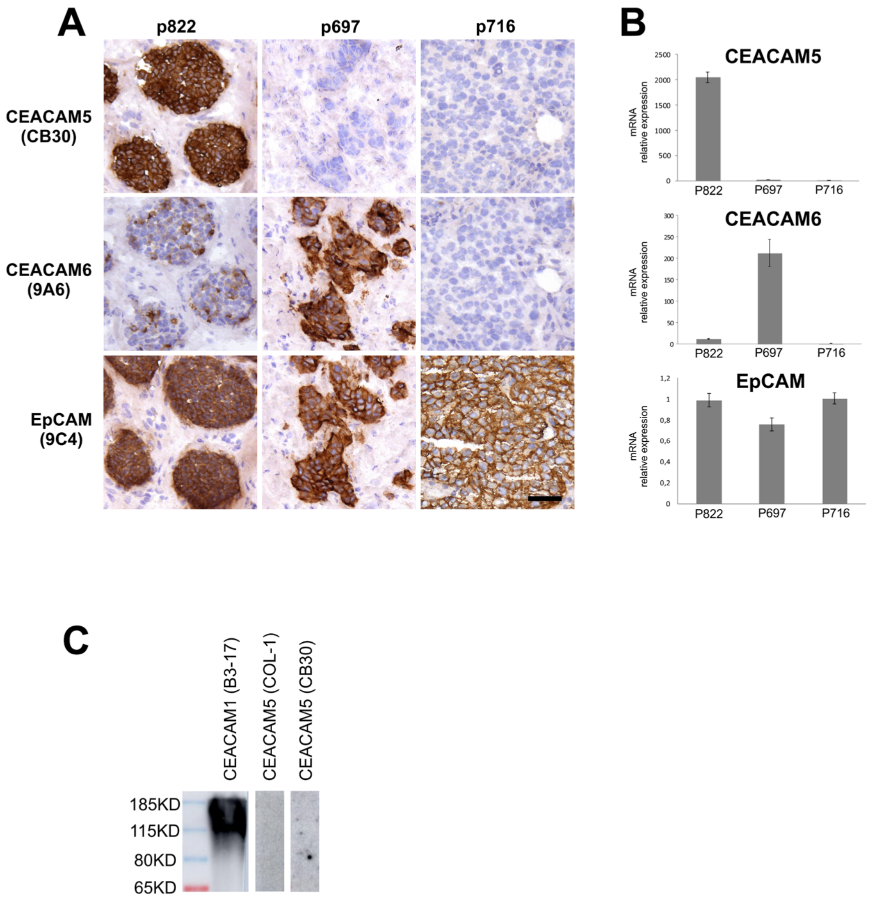

Supplementary Figure 1: Immunohistochemical staining of three different breast carcinomas with CEACAM5 mAb CB30 and CEACAM6 mAb 9A6 correspond to expression levels of mRNA transcripts of *CEACAM5* and *CEACAM6*, respectively. (**A**) Immunohistochemical staining of sections from three breast carcinomas utilizing CEACAM5-specific CB30, CEACAM6-specific 9A6 and EpCAM mAb 9C4. Bar, 50 µm. (**B**) Corresponding expression of transcripts for *CEACAM5*, *CEACAM6* and *EpCAM*. (**C**) Western blot performed on lysate from HEK293 cells overexpressing human CEACAM1 demonstrating that CEACAM5 mAbs CB30 and COL-1 do not cross-react with CEACAM1. CEACAM1 mAb B3-17 was included as a positive control.

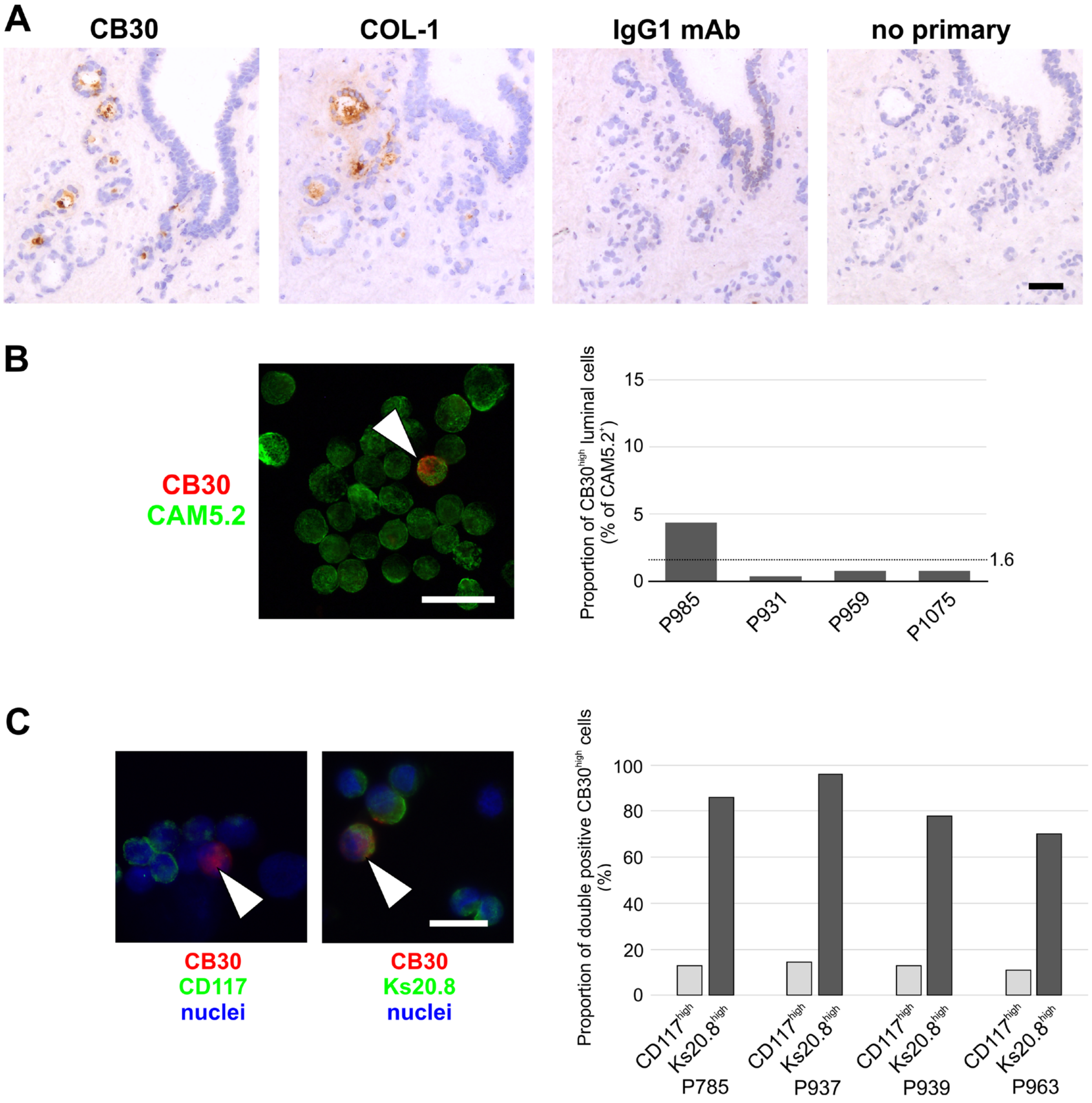

Supplementary Figure 2: CEACAM5 is expressed by a small subset of mature luminal cells in the normal breast gland. (**A**) Serial sections of normal breast stained by immunoperoxidase for CEACAM5 (CB30 and COL-1) as well as an unrelated mAb against PR and a control section with no primary antibody added. Bar, 50 µm. (**B**) Immunofluorescent staining of smears of luminal cells for CEACAM5 (red) and luminal-specific marker CAM5.2 (green) in left panels. Arrowheads mark examples of CEACAM5-positive cells. Bar, 25 µm. Right panel shows graphs of the proportion of CEACAM5-positive cells within the luminal compartment in four biopsies, averaging 1.6% for CEACAM5. For each biopsy, 500 CAM5.2 cells were evaluated. (**C**) Immunosmears doublestained for CEACAM5 (red) and progenitor marker CD117 (green) or mature luminal marker Ks20.8 (green) in left panel. Examples of CEACAM5-positive cells are marked by arrowheads. Bar, 20 µm. Right panel shows the proportion of CEACAM5-positive cells within progenitor and mature luminal compartments in four biopsies. For each biopsy 200 cells were evaluated.

No.	Paired tumor set	Primary tumor	L.N. metastasis
1	M29-1/M29-2	−	−
2	M29-3/M29-4	+/−	+/−
3	M29-5/M29-6	−	−
4	P811/P812	−/+	−/+
5	P835/P836	−/+	−/+
6	P839/P840	+/−	+/−
7	P842/P843♂	−/+	−
8	P845/P846	+/−	+/−
9	P856/P857	+/−	+/−
10	P870/P871	+/−	+/−
11	P942/P943	−	−

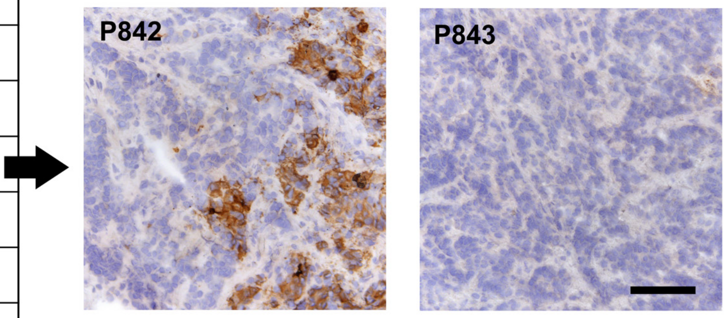

Supplementary Figure 3: Results of immunohistochemical stainings of paired primary breast cancers and corresponding lymph node metastases. Evaluation of 11 sets of cryosectioned breast tumors immunostained for CEACAM5. −: negative; −/+: heterogeneously positive, < 50%; +/−: heterogeneously positive, > 50%. Tumor set marked with ♂ is from a male patient; the other pairs are from female patients. Right panel shows the CEACAM5 staining pattern of the male breast tumor set. Bar, 50 µm.

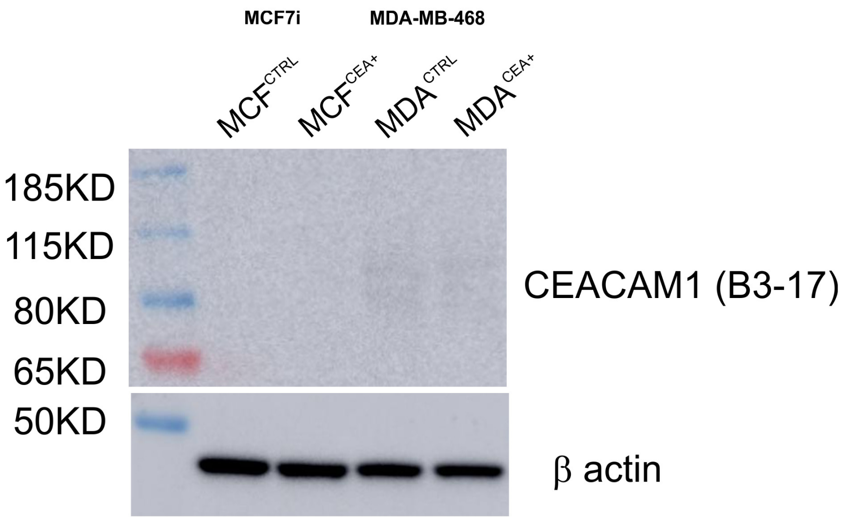

Supplementary Figure 4: Western blot comparison of CEACAM1-expression in MCF7i and MDA-MB468 breast cancer control cells (CTRL) with ectopically CEACAM5-overexpressing sublines (CEA+) demonstrating that CEACAM1 is not induced in CEA+ cells. MAb B-17 was used to analyze CEACAM1 expression while mAb AC-15 against β actin was included as loading control.

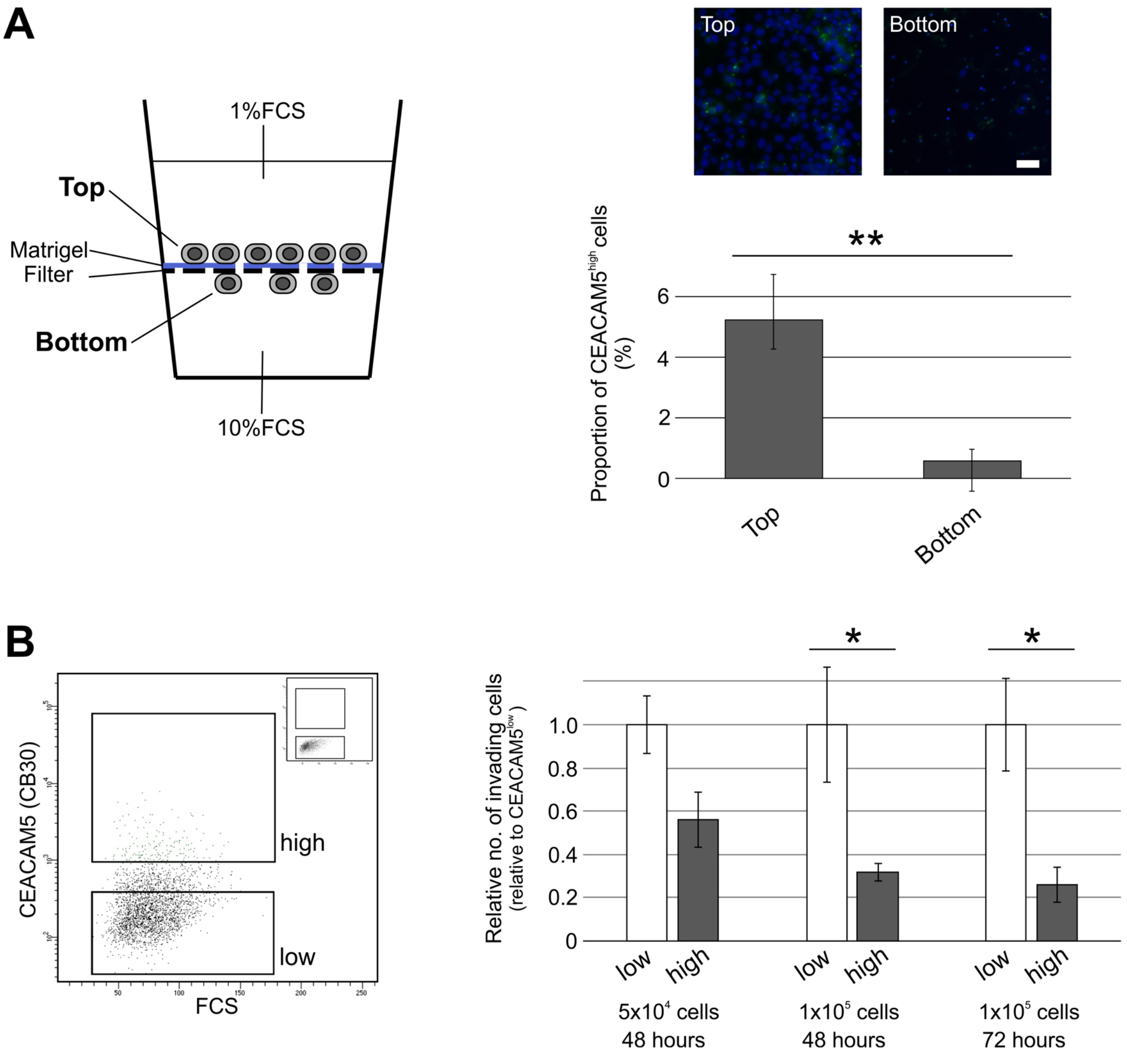

Supplementary Figure 5: Transwell invasion assays demonstrate that CEACAM5-negative cells are more invasive than CEACAM5^{high} cells. (**A**) Left panel is a schematized drawing of the principle of the transwell invasion assay in which cells are placed on top of a filter with 8 µm pores covered with a thin layer of Matrigel. The number of invading cells are assessed on the bottom side of the filters. The right panel shows the proportion of CEACAM5^{high} MCF7i cells in the noninvading versus the invading population in transwell invasion assay. Bar, 50 µm. (**B**) FACS-diagram of MCF7i cells gating for CEACAM5^{high} and CEACAM5$^{neg/low}$ cells in left panel. Insert shows diagram of MCF7i cells with no primary antibody. Right panel shows graphs from three experiments comparing sorted CEACAM5^{high} and $^{neg/low}$ cells in the transwell invasion assay. * and ** indicate $p < 0.05$ and $p < 0.01$ tested by t test, respectively.

Supplementary Table 1: Evaluation of 110 breast carcinomas stained by immunohistochemistry, divided into molecular profiles Luminal A, Luminal B, HER2-enriched and Triple-negative

Biopsy	CEA	CEA	CEA	CEACAM6
	CB30	COL-1	1105	9A6
		**Luminal A**		
P696	+[d]	+[d]	+[d]	+[d]
P701	–[b]	–	+[d]	+[d]
P703	–	–	–	–
P707	–[b]	–[b]	+[d]	+[c]
P720	+[c]	+[c]	+[e]	+[e]
P721-B	**–[b]**	**–**	+[d]	+[d]
P726-1	+[d]	+[d]	+[d]	+[d]
P760-1	+[c]	+[c]	+[d]	+[c]
P761	+[d]	+[d]	+[e]	+[e]
P775	+[c]	+[c]	+[d]	+[d]
P786	+[c]	+[c]	+[d]	+[d]
P787	+[c]	+[c]	+[e]	+[e]
P788	+[e]	+[e]	+[e]	+[e]
p822	+[e]	+[e]	+[e]	+[d]
P835*	+[d]	+[d]	+[e]	+[d]
P870*	+[e]	+[e]	+[e]	+[e]
P874	+[e]	+[e]	+[e]	+[e]
P875	+[d]	+[d]	+[e]	+[e]
P883	–	–	–	–
P887	+[e]	+[e]	+[e]	+[e]
P891	+[d]	+[d]	+[e]	+[e]
P919	+[d]	+[d]	+[e]	+[e]
P929	**+[d]**	**+[e]**	+[e]	+[e]
P947	–	–	+[d]	+[d]
P953	+[e]	+[e]	+[e]	+[e]
P989	–[b]	–[b]	+[d]	+[c]
P991	+[c]	+[c]	+[c]	–
P1031	–	–	–	–
P1033	+[e]	+[e]	+[e]	+[e]
P1035	–[b]	–[b]	+[c]	+[c]
P1036	–	–	+[e]	+[e]
P1041	+[d]	+[d]	+[e]	+[e]
P1048	–	–	–	–
P1051	–[b]	–[b]	+[c]	+[c]
P1054	+[c]	+[c]	+[d]	+[d]
P1059	+[d]	+[d]	+[e]	+[d]
P1061	+[c]	+[c]	+[e]	+[e]
P1062	–	–	–	–
P1066	+[c]	+[c]	+[e]	+[e]
P1068	+[e]	+[e]	+[e]	+[e]
P1099	+[d]	+[d]	+[d]	–[b]
P1100	+[e]	+[e]	+[e]	+[e]
P1101	–	–	+[e]	+[e]
P1102	+[d]	+[d]	+[d]	–[b]
P1103	+[c]	+[c]	+[e]	+[d]
P1105	+[d]	+[d]	+[e]	+[d]

P1106	+[d]	+[d]	+[d]	+[d]
P1111	–	–	–	–
P1112	+[e]	+[e]	+[e]	+[e]
Total	34/49	34/49	43/49	40/49
**Luminal B**				
w275-4	+[d]	+[d]	+[e]	+[d]
w354-4	+[e]	+[e]	+[e]	+[e]
P716	–	–	–	–
P807	+[d]	+[d]	+[d]	+[d]
P834-1	+[e]	+[e]	+[e]	+[d]
P892	+[e]	+[e]	+[e]	+[e]
P917	+[e]	+[e]	+[e]	+[e]
P1040	–	–	+[d]	+[d]
P1055	–	–	–[b]	–[b]
P1063	–	–	–[b]	–
W3096-10	+[c]	+[c]	+[d]	+[d]
Total	7/11	7/11	8/11	8/11
**HER2-enriched**				
w164-1	+[c]	+[c]	+[e]	+[e]
P736	–	–	+[c]	+[c]
P764	–[b]	–[b]	+[e]	+[e]
P811*	+[c]	+[c]	+[e]	+[e]
P839*	+[e]	+[e]	+[e]	+[e]
P886	–	–	–	–
P888	+[e]	+[e]	+[e]	+[e]
P897	–[b]	–[b]	+[e]	+[e]
P901	+[e]	+[e]	+[e]	+[e]
P907	+[d]	+[d]	+[e]	+[e]
P918	+[d]	+[d]	+[e]	+[e]
P950	+[d]	+[d]	+[e]	+[e]
P1067	+[d]	+[d]	+[e]	+[d]
W1872-2	+[e]	+[e]	+[e]	+[e]
W1872-3	+[e]	+[e]	+[e]	+[e]
W3175	+[d]	+[d]	+[d]	+[d]
Total	12/16	12/16	15/16	15/16
**Triple-negative**				
M29-5*	–	–	–	–
W117-1	+[d]	+[d]	+[e]	+[e]
W190-1	–	–	–	–
W355-2	–	–	–	–
P687	+[d]	+[d]	+[d]	+[d]
P697	–	–	+[e]	+[d]
P709	–	–	–	–
P757	–[b]	–[b]	+[d]	+[d]
P766	–	–	–	–
P783	–	–	–	–
P853	–	–	–	–
P860	+[e]	+[e]	+[e]	+[e]
P873	**+[e]**	**+[d]**	+[e]	+[e]
P905	–	–	–	–
P909	–	–	–	–

P914	–	–	–	–
P921	–	–	–	–
P924	–	–	+[e]	+[e]
P942*	–	–	–	–
P949	–	–	–	–
P1044	+[e]	+[e]	+[e]	+[e]
P1049	–	–	–	–
P1052	–	–	–	–
P1056	–	–	–	–
P1060	–	–	–	–
P1065	–	–	–	–
W1076-5	–	–	–	–
P1104	–	–	–[b]	–[b]
W1731-3	+[c]	+[c]	+[c]	+[c]
W1884-1	+[d]	+[d]	+[d]	+[d]
W1884-3	–	–	–	–
W3096-9	–	–	+[c]	+[c]
W3141-6	**+[e]**	**+[d]**	+[e]	+[e]
W3147-1	–	–	+[d]	+[d]
Total	8/34	8/34	13/34	13/34

Tumors were stained with antibodies against CEACAM5 (clones CB30 and COL-1), CEACAM5/6 (clone 1105) and CEACAM6 (clone 9A6). Tumors were considered negative when no (–) or < 1% positive neoplastic cells (–[b]) were detected. Positive tumors were subdivided into < 10% positive cells (+[c]), < 50% positive cells (+[d]) and > 50% positive cells (+[e]). Minor discrepancies between mAbs CB30 and COL-1 are marked in Bold. Tumors marked with *were also included in the primary tumor and lymph node metastases set in Supplementary Figure 3.

Supplementary Table 2: Relapse-free survival of breast cancer groups trichotomized into patients with CEACAM5^{low} expression (T1) and CEACAM5^{high} expression (T3)

	No. of patients in test			Relapse-free Survival	
**Group**	**Total**	**T1**	**T3**	***P* value**	**HR**
All	3951	1310	1341	0.2874	0.93 (0.81–1.06)
**ER-positive**	**2061**	**683**	**701**	**0.0023**	**1.39 (1.12–1.71)**
ER-negative	801	264	272	0.9	1.02 (0.77–1.35)
Luminal A	1933	639	657	0.1958	1.15 (0.93–1.43)
Luminal B	1149	379	391	0.5842	1.07 (0.85–1.34)
HER2	251	83	85	0.628	0.89 (0.55–1.43)
**Basal-like***	**618**	**208**	**209**	**0.025**	**0.71 (0.53–0.96)**
**Basal-like 1***	**171**	**56**	**58**	**0.0063**	**0.44 (0.24–0.81)**
**Luminal androgen receptor***	**203**	**67**	**69**	**0.0116**	**0.51 (0.3–0.87)**
Mesenchymal*	177	59	60	0.0867	0.65 (0.39–1.07)
Immunomodulatory*	203	67	69	0.5165	1.3 (0.58–2.92)
Basal-like 2*	76	25	26	0.565	0.77 (0.31–1.89)
Mesenchymal stem-like*	63	21	21	0.9312	1.05 (0.38–2.89)

Values were calculated at Kaplan-Meier Plotter (https://kmplot.com) utilizing JetSet best probe set 201884_at with a follow up threshold set at 120 months. Statistically significant groups are highlighted in Bold. *marks subsets that are considered subgroups of TN breast cancer.

Supplementary Table 3: Overall survival of breast cancer groups trichotomized into patients with CEACAM5^{low} expression (T1) and CEACAM5^{high} expression (T3)

	No. of patients in test			Overall survival	
**Group**	**Total**	**T1**	**T3**	***P* value**	**HR**
**All**	**1402**	**463**	**477**	**0.0192**	**1.38 (1.05–1.8)**
ER-positive	548	181	186	0.0648	1.54 (0.97–2.45)
ER-negative	251	83	85	0.6411	1.14 (0.65–1.99)
Luminal A	611	203	206	0.293	1.27 (0.81–1.98)
**Luminal B**	**433**	**143**	**147**	**0.0013**	**2.12 (1.33–3.38)**
HER2	117	39	40	0.5103	0.75 (0.33–1.75)
Basal-like	241	80	81	0.43	1.27 (0.7–2.33)

Values were calculated at Kaplan-Meier Plotter (https://kmplot.com) utilizing JetSet best probe set 201884_at with a follow up threshold set at 120 months. Statistically significant groups are highlighted in Bold.
